# The association between serum vitamin D levels and renal tubular dysfunction in a general population exposed to cadmium in China

**DOI:** 10.1371/journal.pone.0195682

**Published:** 2018-04-10

**Authors:** Xiao Chen, Yan Dai, Zhongqiu Wang, Guoying Zhu, Xiaoqiang Ding, Taiyi Jin

**Affiliations:** 1 Department of Nephrology, Zhongshan Hospital Fudan University, Shanghai Key Laboratory of kidney and dialysis, Shanghai, China; 2 Department of Radiology, Affiliated Hospital of Nanjing University of Chinese Medicine, Nanjing, China; 3 Institute of Radiation Medicine, Fudan University, Shanghai, China; 4 Department of Occupational Medicine, School of Public Health, Fudan University, Shanghai, China; "INSERM", FRANCE

## Abstract

Cadmium exposure can cause renal tubular dysfunction. Recent studies show that vitamin D can play multiple roles in the body. However, the association between serum vitamin D levels and renal tubular dysfunction in a general population exposed to cadmium has not been clarified. We performed study to assess the effects of cadmium on serum 25(OH) D levels and the association between serum 25(OH) D levels and renal tubular dysfunction in a population environmentally exposed to cadmium. A total of 133 subjects living in control area and two cadmium polluted areas were included in the present study. Cadmium in urine (UCd) and blood (BCd), urinary β2Microglobulin (UBMG), urinary retinol binding protein (URBP) and serum 25 (OH) D were determined. Logistic regression was used to estimate the association between 25 (OH) D and prevalence of renal tubular dysfunction. No significant differences were observed in serum 25(OH) D levels among the four quartile of UCd and BCd after adjusting with cofounders. After adjusted with the confounders, the odds ratio (OR) of subjects with 25(OH) D ≥ 40 ng/ml were 0.20 (95%CI: 0.1–0.8) if UBMG was chosen as indicators of renal dysfunction and 0.28 (95%CI: 0.1–1.1) if URBP was chosen as indicators of renal dysfunction, compared with those with 25(OH) D < 30 ng/ml, respectively. Similar results were observed in those subjects living in cadmium polluted areas or with high level of UCd or BCd. Our data indicated that cadmium exposure did not affect serum 25(OH) D level and high 25 (OH) D levels were associated with a decreased risk of renal tubular dysfunction induced by cadmium.

## Introduction

Cadmium is one common harmful heavy metal that is widely distributed in our environment. It is still a major concern for public health because accumulating epidemiological studies have shown the associations between environmental level of cadmium exposure and adverse health risks [1, 2]. Food and smoking are the two main way for cadmium exposure in general population. Cadmium exposure can cause several adverse effects or diseases, including renal dysfunction, liver damage, cardiovascular disease, and osteoporosis [3]. Kidney is one of the important target organs for cadmium toxicity. Cadmium exposure is associated with high risk of renal tubular dysfunction or chronic kidney disease (CKD) [1, 4, 5].

Several previous population studies showed that serum zinc was statistically associated with the risk of cadmium nephrotoxicity [6, 7] which indicated that nutritional status may influence the cadmium-induced kidney damage. Vitamin D is also a biomarker of nutritional status. Vitamin D is required for calcium absorption in intestines and is necessary for bone metabolism [8]. However, recent studies have deeply changed our view on the biological function of vitamin D [9]. Vitamin D can play multiple roles in the body, such as regulating cell growth, immune function, inflammatory reaction and antitumorogenic activity [8, 10]. The roles of vitamin D in respiratory disease [11, 12], diabetes mellitus (DM) [13], cardiometabolic disease, multiple sclerosis [14] and cancer [15] have been investigated. Recent studies also demonstrate that high free and bioactive vitamin D levels are associated with lower risk of end-stage renal disease [16] and vitamin D is essential for maintaining the stability of proximal tubular epithelial monolayer in uremic conditions [17]. Previous studies also show that active form of vitamin D, paricalcitol, can reduce renal inflammation in patients with CKD [18] and DM [19]. However, the association between vitamin D level and renal dysfunction in the general population that exposed to cadmium has not been clarified.

Cadmium exposure may result in decrease of 25 (OH) D_3_ [20]. However, those effects were not observed in several studies [21, 22]. In the present study, we showed the effects of cadmium exposure on serum 25 (OH) D_3_ levels in a Chinese community-based population. Moreover, we investigated the association between serum vitamin D levels and renal tubular dysfunction.

## Materials and method

### Study area and population

Two cadmium polluted areas and a control area located at the southeast China were included in this study. A smelter began production from 1961 was located at the heavily polluted area. The waste water from the smelter was directly discharged into nearby river until 1995. The local residents used the river water to irrigate their fields. Our previous studies showed that average cadmium in rice was 3.7 mg/kg. Another area, 12 km away from the smelter, was selected as the moderate polluted area. The cadmium in rice was 0.5 mg/kg. An area 40 km away from the smelter was chosen as the control area. The subjects consume the commercial rice (cadmium concentration = 0.07 mg/kg). The dietary pattern, living conditions, social and economic status were similar among the three areas.

Those subjects who were born in the three areas and consumed locally grown rice throughout their entire lifetime were included in this study. 20 families were randomly selected in each area (152 subjects). 12 subjects were absent in their home at the time of investigation. Seven subjects with unknown liver dysfunction, CKD, and DM were excluded. Finally, a total of 133 subjects, 36 men and 97 women were finally included in the present study. All participants completed a questionnaire, including demographic information, cigarette smoking, alcohol consumption and medical history. The study protocol and consent procedure were approved by Ethics Committee of Fudan University, China. Written informed consent was obtained from each participate.

### Cadmium determination

Cadmium in urine (UCd) and blood (BCd) were determined as our previous study described. Briefly, urine samples were collected using container that was soaked with HNO_3_ solution (5%) and then washed with deionized water. 5 ml blood sample was collected without anticoagulant and centrifuged for serum isolation. 2 ml blood sample was collected with anticoagulant for cadmium assay. All urine and blood samples were stored at -20°C in local laboratory and stored at -80° C in our institution until analyzed. UCd and BCd were determined by using graphite-furnace atomic absorption spectrometry (GF-AAS) as our previous study described [23, 24]. Strict procedure for quality control was performed during the cadmium determination [23, 24].

### Renal biomarkers determination

Several biomarkers reflecting with renal function were measured: urinary creatinine (UCr), urinary β_2_Microglobulin (UBMG) and urinary retinol binding protein (URBP). Briefly, UBMG was assayed by radioimmunosorbent assay (RIA); UALB was measured by enzyme-linked immunosorbent assay (ELISA); UCr was determined by the Jaffe reaction method. All urinary markers, including UCd, UBMG and URBP, were adjusted with UCr.

### 25 (OH) D determination

Serum 25(OH) D was analyzed using ELISA kit (IDS, UK). Serum samples were processed in accordance with manufacturer’s instructions. The inter-assay and intra-assay CVs were both lower than 10.0%.

### Statistical analysis

Statistical analysis was performed by using SPSS 16.0 (SPSS Inc., Chicago, IL, USA). Data was shown as mean with standard deviation or median with 95% confidential interval (CI). Serum 25 (OH) D was divided into three level: < 30 ng/ml, 30–40 ng/ml and ≥ 40 ng/ml. One way ANNOVA, ANCOVA or Chi-square test was used for statistical analysis. UCd and BCd were categorized based on their quartile distribution (<25th percentile, 25-50th percentile, 50-75th percentile and ≥75th percentile). Spearman correlation was used to show the association between serum 25 (OH) D levels and renal effect biomarkers. Logistic regression models were used to show the association between prevalence of renal dysfunction and serum 25 (OH) D levels. P < 0.05 was considered statistically significant.

## Result

### Characteristics of study population

The characteristics of subjects are list in Table 1. All subjects were divided into three groups according to their 25 (OH) D levels. There were no significant differences in age, height, weight, gender, drinking and smoking habits among these three groups. Similar results were observed in UCd, BCd and URBP. The UBMG in those subjects with the highest level of 25 (OH) D was significant lower than those subjects with the lowest 25 (OH) D (p = 0.02).

**Table 1:** Characteristics of subjects.

	25(OH)D levels			
	<30 (n = 53)	30–40 (n = 43)	≥40 (n = 37)	p
Age	56.8±11/9	54.3±13.2	56.5±10.7	>0.05
Height	155.2±7.6	156.7±9.2	153.7±7.4	>0.05
Weight	56.8±11.9	54.6±8.7	54.5±8.5	>0.05
Gender(male)	14	12	10	>0.05
Smoking	7	6	6	>0.05
Drink	8	10	7	>0.05
BCd(µg/L)	12.1(8.4–16.8)	10.1(7.5–12.9)	7.8(4.6–11.8)	>0.05
UCd(µg/g cr)	12.6(9.3–16.5)	10.0(7.2–12.8)	9.6(6.0–14.0)	>0.05
UBMG(mg/g cr)	0.69(0.4–1.1)	0.88(0.33–1.6)	0.52(0.17–1.0)^*^	>0.05
URBP(mg/g cr)	0.34(0.2–0.6)	0.62(0.1–1.6)	0.3(0.1–0.6)	>0.05

*p = 0.02 vs < 30 ng/ml

UCd: cadmium in urine; BCd: cadmium in blood; UBMG: urinary β_2_Microglobulin; URBP: urinary retionl binding protein.

Age, weight, height and BMI are shown as mean value ± standard deviation.

UCd, BCd, UBMG and URBP are shown as median (95% confidence intervals).

### Serum 25 (OH) D at different UCd and BCd levels

**Fig 1:**
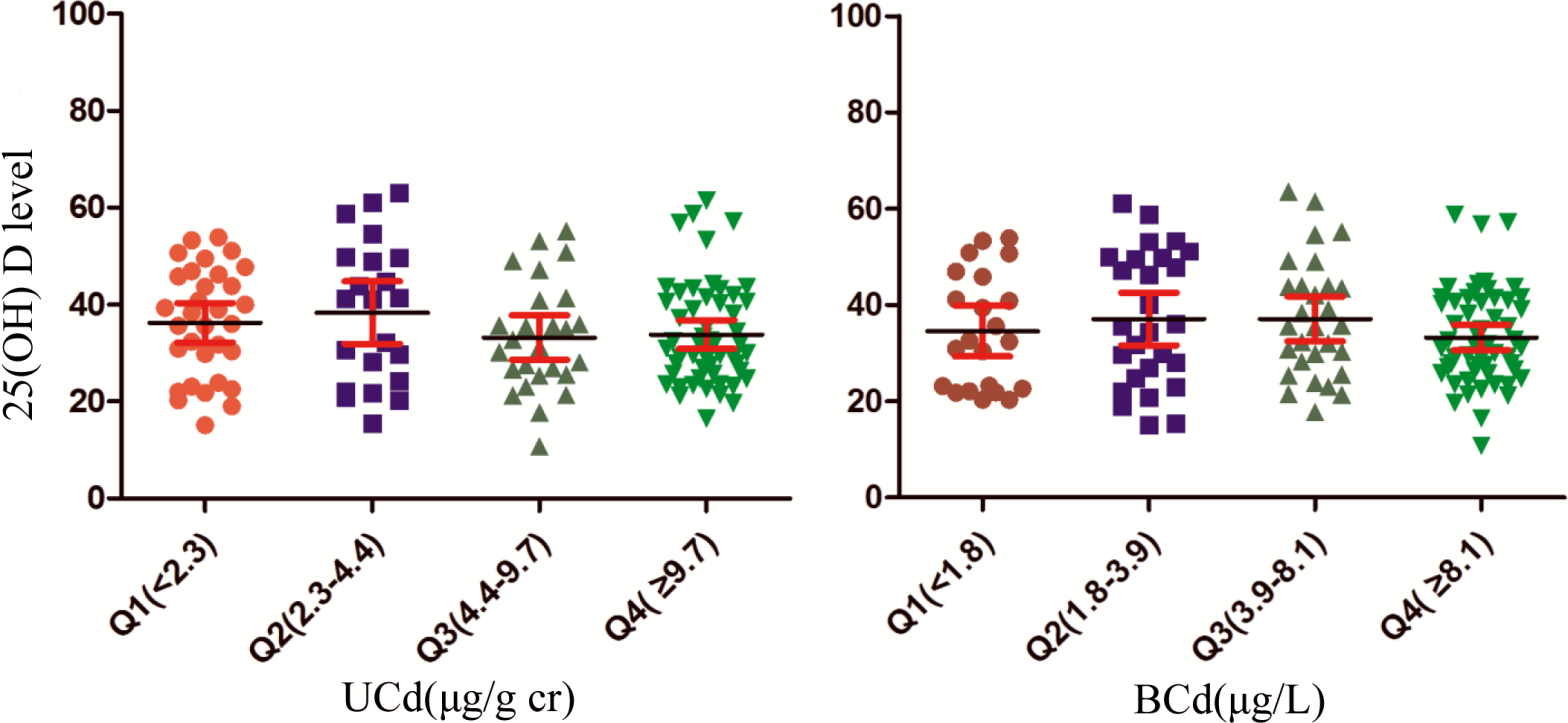
The serum 25(OH) D at different UCd and BCd levels. UCd and BCd were categorized based on their quartile distribution (<25th percentile, 25-50th percentile, 50-75th percentile and ≥75th percentile). UCd: cadmium in urine; BCd: cadmium in blood.

**Fig 2:**
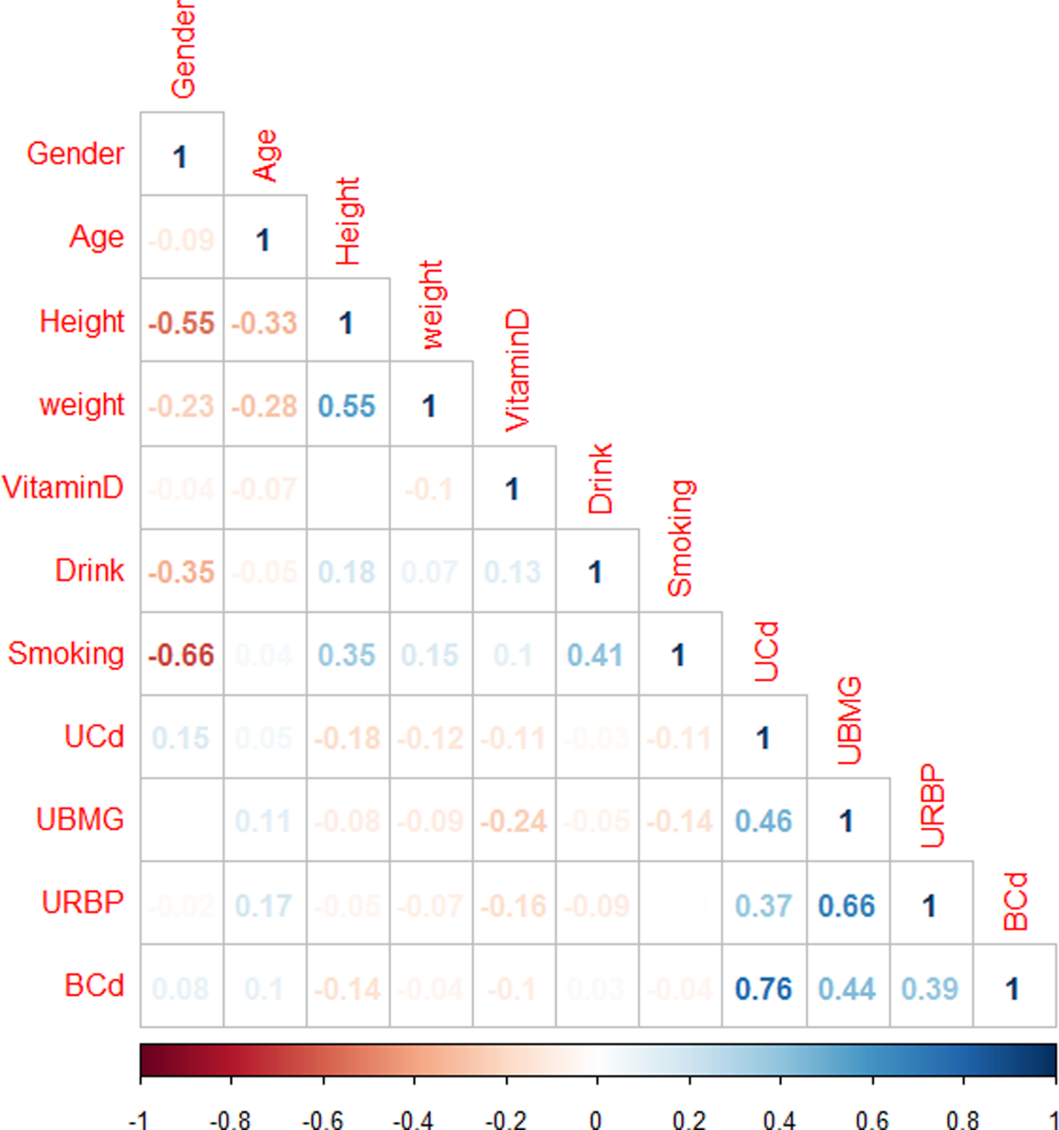
Spearman correlation analyses. Serum 25 (OH) D levels were not correlated with UCd or BCd, but negatively associated with UBMG and URBP. UCd, cadmium in urine; BCd: cadmium in blood; UBMG: urinary β2Microglobulin; URBP: urinary retinol binding protein.

No significant differences were observed in serum 25(OH) D levels among the four quartile of UCd and BCd (Fig 1). Similar results were observed after adjusting with confounders, including age, sex and weight. Correlation analysis also indicated that there was no obvious association between serum 25 (OH) D levels and UCd or BCd (Fig 2).

### Renal dysfunction and serum 25 (OH) D in total population

Subsequently, we analyzed the association between serum 25 (OH) D and prevalence of renal dysfunction in total population. Spearman correlation analysis (Fig 2) showed that serum 25 (OH) D levels were negatively associated with UBMG and URBP (p < 0.05).

The odds ratio (OR) of subjects with 25(OH) D ≥ 40 ng/ml are 0.17 [95% confidence interval (CI): 0.1–0.6] if UBMG was chosen as indicator of renal dysfunction and 0.20 (95%CI: 0.1–0.8) if URBP was chosen as indicator of renal dysfunction, compared with those with 25 (OH) D < 30 ng/ml (Table 2). After adjusted with the confounders, the odds ratio were 0.20 (95%CI: 0.1–0.8) and 0.28 (95%CI: 0.1–1.1), respectively.

**Table 2:** Odds ratios (ORs) and 95% confidence intervals (CIs) of renal dysfunction and serum 25 (OH) D in total population.

	Odds ratio (95%CI)		
	25(OH)D	Model 1	Model 2
UBMG	Reference (<30)	1	1
	30–40	0.55(0.2–1.5)	0.56(0.2–2.5)
	≥40	0.17(0.1–0.6)	0.20(0.1–0.8)
URBP	Reference(<30)	1	1
	30–40	0.67(0.2–2.0)	0.84(0.2–3.0)
	≥40	0.20(0.1–0.8)	0.28(0.1–1.1)

UBMG: urinary β_2_Microglobulin; URBP: urinary retionl binding protein.

Model 1, adjusted for age, sex, height and weight; Model 2, adjusted for age, sex, height, weight, smoking and alcohol habits, cadmium in blood (BCd) and urinary cadmium (UCd).

### Renal dysfunction and serum 25(OH) D in subjects living in cadmium-polluted areas

Subsequently, we analyzed the association between serum 25 (OH) D and prevalence of renal dysfunction in subjects living in cadmium-polluted areas by using logistic regression model. UBMG and URBP were chosen as indicators of renal dysfunction. The odds ratio (OR) of subjects with 25(OH) D ≥ 40 ng/ml were 0.17 (95%:0.05–0.6) and 0.23 (95%CI: 0.1–0.9) compared with those with 25 (OH) D < 30 ng/ml, respectively (Table 3). After adjusted with the confounders, the odds ratio were 0.22 (95%CI: 0.1–0.9) and 0.37 (95%CI: 0.1–1.6), respectively.

**Table 3:** Odds ratios (ORs) and 95% confidence intervals (CIs) of renal dysfunction and serum 25 (OH) D in population living in polluted areas.

	Odds ratio (95%CI)		
	25(OH)D	Model 1	Model 2
UBMG	Reference (<30)	1	1
	30–40	0.48(0.2–1.4)	0.55(0.1–2.2)
	≥40	0.17(0.05–0.6)	0.22(0.1–0.9)
URBP	Reference (<30)	1	1
	30–40	0.88(0.3–2.9)	1.15(0.3–4.5)
	≥40	0.23(0.1–0.9)	0.37(0.1–1.6)

UBMG: urinary β_2_Microglobulin; URBP: urinary retionl binding protein.

Model 1, adjusted for age, sex, height and weight; Model 2, adjusted for age, sex, height, weight, smoking and alcohol habits, cadmium in blood (BCd) and urinary cadmium (UCd).

### Renal dysfunction and serum 25 (OH) D in subjects with high BCd or UCd

Furthermore, we evaluated the association of serum 25 (OH) D and renal dysfunction in subjects with high levels of UCd (≥3.0 μg/g cr) (Table 4) and BCd (≥ 2.0 μg/L) (Table 5). For those subjects with high level of UCd, the odds ratio (OR) of subjects with 25 (OH) D ≥ 40ng/ml are 0.19 (95%:0.05–0.7) if UBMG was selected as indicator of renal dysfunction. Similar result was observed after adjusting with the confounders (OR = 0.23, 95%CI: 0.1–0.9). The risk of renal tubular dysfunction was also decreased in subjects with the highest level of 25 (OH) D if URBP was chosen as indicator, but no statistical significance was found.

**Table 4:** Odds ratios (ORs) and 95% confidence intervals (CIs) of renal dysfunction and serum 25 (OH) D in population with UCd ≥ 3.0 μg/g cr.

	Odds ratio (95%CI)		
	25(OH) D	Model 1	Model 2
UBMG	Reference (<30)	1	1
	30–40	0.48(0.2–1.5)	0.61(0.1–2.5)
	≥40	0.19(0.05–0.7)	0.23(0.1–0.9)
URBP	Reference (<30)	1	1
	30–40	0.81(0.2–2.7)	1.08(0.3–4.3)
	≥40	0.31(0.1–1.4)	0.43(0.1–1.8)

UBMG: urinary β_2_Microglobulin; URBP: urinary retionl binding protein.

Model 1, adjusted for age, sex, height and weight; Model 2, adjusted for age, sex, height, weight, smoking and alcohol habits, cadmium in blood (BCd) and urinary cadmium (UCd).

**Table 5:** Odds ratios (ORs) and 95% confidence intervals (CIs) of renal dysfunction and serum 25 (OH) D in population with BCd ≥ 2.0 μg/L.

	Odds ratio (95%CI)		
	25(OH)D	Model 1	Model 2
UBMG	Reference (<30)	1	1
	30–40	0.54(0.2–1.6)	0.53(0.1–2.1)
	≥40	0.19(0.05–0.7)	0.19(0.1–0.8)
URBP	Reference (<30)	1	1
	30–40	0.86(0.3–2.7)	0.93(0.2–3.5)
	≥40	0.22(0.05–0.9)	0.28(0.1–1.1)

UBMG: urinary β_2_Microglobulin; URBP: urinary retionl binding protein.

Model 1, adjusted for age, sex, height and weight; Model 2, adjusted for age, sex, height, weight, smoking and alcohol habits, cadmium in blood (BCd) and urinary cadmium (UCd).

For those subjects with high level of BCd, the two logistic models both showed that the risk of renal tubular dysfunction was decreased if UBMG was chosen as indicator of renal dysfunction (OR = 0.19, 95%CI: 0.05–0.7; OR = 0.23 (95%CI: 0.1–0.9, respectively). Similar results were observed if URBP was chosen as indicator, but no statistical significance was found after adjusting with confounders.

## Discussion

Vitamin D is usually known as a nutrient which is critical for growth and remodeling of bone. However, recent more and more studies indicate that vitamin D has a broader spectrum of its activity [25], such as regulating inflammatory reaction and antitumorogenic activity. Cadmium exposure can cause renal damage. However, the associations between vitamin D level and renal dysfunction caused by cadmium exposure have not been completely clarified. In the present study, based on a community-based population, we observed that high 25 (OH) D levels were associated with a lower risk of renal tubular dysfunction induced by cadmium compared with those with insufficient vitamin D. Sufficient vitamin D may have a beneficial effects on cadmium-induced renal dysfunction.

The exposure to cadmium may lead to decrease of 25 (OH) D [20] in rat model. However, those effects were not observed in several studies [21, 22, 26]. In our study, no significant correlation was observed between serum 25(OH) D and UCd or BCd, which was consistent with the previous population study [26]. The major natural source of the vitamin D_3_ is from skin, where the cholesterol is converted into cholecalciferol through a sequential chemical reaction in the presence of sun exposure [27]. Cholecalciferol is converted into calcifediol (25-hydroxyvitamin D) by the liver. High level of cadmium can induce liver damage. Therefore, cadmium exposure may lead to decrease of 25 (OH) D. However, the low level of cadmium exposure cannot induce severe liver dysfunction. Consequently, low level of cadmium exposure may not affect serum 25 (OH) D levels. Cadmium in kidney may be a better biomarker than UCd or BCd. X-ray fluorescence is one of useful approaches in the determination of metals in organs [28, 29]. However, there are no indications to perform the invasive biopsy in our population. Therefore, the association between vitamin D and cadmium in kidney was not evaluated.

Previous data have shown that 1, 25 (OH) vitamin D_3_ can decrease albuminuria in nephritis in animal models [30, 31]. However, due to the short half-life of 1, 25 (OH) vitamin D_3_ in blood, levels of 25 (OH) D are usually regarded as the vitamin D status [16]. Recent studies further indicated that 25 (OH) D deficiencies were related to the severe reduction of eGFR in CKD patients [32, 33] and high risk of incident ESRD [16]. 25 (OH) vitamin D deficiencies were also associated with poor clinical outcomes of IgA nephropathy [34] and non-dialysis-dependent CKD [35, 36]. Many epidemiological studies indicate that environmental level of cadmium exposure increases the risk of renal dysfunction [1, 4]. Animal studies also showed that vitamin D deficiency enhanced the cadmium toxic effects to kidney [37]. However, human data is limited. Our data demonstrated that 25 (OH) vitamin D insufficiencies were associated with high risk of cadmium-induced renal tubular dysfunction. Sufficient vitamin D may protect against cadmium-induced kidney damage. However, several studies also indicate that combination of high calcium and vitamin D supplementation increase the risk of kidney stone in humans [38] and animal model [39]. We did not evaluate the association between vitamin D level and kidney stone in our study. There are no additional calcium and vitamin D supplementation in our population. Therefore, high vitamin D level may be not increase the risk of kidney stone or tissue calcifications.

The molecular mechanism of vitamin D in kidney diseases is still not clarified. Its activity on immune function and inflammatory reaction may play a critical role. Gonçalves et al. [40] indicated that 25 (OH) D deficiencies might enhance tubulointerstitial damages, such as fibrosis and inflammatory infiltration, through those inflammatory pathways. In addition, vitamin D may improve the oxidative stress and inflammatory reaction [25], which is one of important pathological mechanism of renal injury [41,42]. Cadmium also induces renal damage via inflammatory pathways and oxidative stress [43–45]. Therefore, the protected effects of vitamin D against cadmium-induced renal dysfunction may be due to its role on oxidative stress and inflammatory reaction. In addition, several clinical trials showed that vitamin D supplementation may improve the vascular status in CKD [46, 47].

One main strength of our study is that we analyze the associated between renal tubular dysfunction and cadmium exposure in several sub-populations, including those population living in polluted areas and those subjects with high levels of UCd and BCd. Interestingly, similar results were observed in those several sub-groups. The risk of renal dysfunction was all statistically decreased (77%-81%) in subjects with high level 25 (OH) D compared with those with 25 (OH) D insufficiencies. Another strength is that the blood samples are collected within one week at same season which diminishes the influence of season on serum 25 (OH) D.

There are also several limitations in our study. First, only 133 subjects were included in this study. The population size was relatively small. Second, we did not observe the gender differences in the association between 25 (OH) D and cadmium-induced renal dysfunction due to the small size of male population. Third, we cannot exclude some unknown confounding factors that may influence our results, such as zinc and calcium intake from food. In addition, the overall 25 (OH) D was determined in this study, and we did not know the association between free or bioactive 25(OH) D or active vitamin D and cadmium-induced renal damages.

In conclusion, our data showed that cadmium exposure does not affect the serum 25(OH) D levels. Moreover, we observe that high level of 25 (OH) D body burden is associated with lower risk of cadmium-induced renal tubular dysfunction. Our study indicates that 25 (OH) D supplement use may protect against cadmium-induced tubular damage in humans.

## Supporting information

S1 File Questionnaire. (PDF)

## Abbreviations

BCd: cadmium in blood
CI: confidential interval
CKD: chronic kidney disease
DM: diabetes mellitus
ELISA: enzyme-linked immunosorbent assay
GF-AAS: graphite-furnace atomic absorption spectrometry
OR: odds ratio
UBMG: urinary β2Micorglobulin
UCd: Cadmium in urine
UCr: urinary creatinine
URBP: urinary retinol binding protein
