# Supplementary material for: The association between serum vitamin D levels and renal tubular dysfunction in a general population exposed to cadmium in China
Source: PLoS One. 2018 Apr 10;13(4):e0195682. doi: 10.1371/journal.pone.0195682 (PMC5892922; doi:10.1371/journal.pone.0195682)
Supplement: S1 File — (PDF) [file pone.0195682.s001.pdf]

QESTIONNAIRE

一. BACKGROUND

{num}code#### {BMD} bone density#.###
{ucd}urinary cadmium(ug/l) {ucdcr} urinary cadmium adjusted by creatinine(ug/g.cr)
{cr} creatinine(g/l)
{h}eight (cm) ###. {w}eight (Kg) ##.#
{name}______ {x2} Unit ##
{age} ##
{sex}:(1)male (2)female #
{educa}tion: (0) illiterate (1) primary (2) middle (3) high school (4) master or higher #
{marry} (0)unmarriage (1)divorced (2)married (3)remarriage(4)widow #
{local} Are you born in this village?(1)yes (0)no #
{intime} When did you immigrate into this villiage? <mm/dd/yy>
{occu}pational history #

Hazard Factor :
{ tbegin1} When did you begin to work <mm/dd/yy>
{tend1} When did you end this work <mm/dd/yy>
{unit1} company ##
{type1} worktype ##
{factor11} exposure hazards ##
{factor12} ## {factor13} ## {factor14} ## {factor15} ##
{hour1} exposure time(h/day) ##
{level1} exposure level(mg/m3) ####

{smoke}Are you smoking? (0) no (1) yes (2) giveing up #
{tsquit} when quit smoking <mm/dd/yy>
{tbegin} when start to smoke <mm/dd/yy>
{stake} how many sticks you take per day(sticks/day) ##
{stype} cigarette type: (0) imported (1) dosmetic #
{drink}ing alcohol? (0) Never (1) occasionaly (2) often (3) quit #
{tdrink} when start to take alcohol <mm/dd/yy>
{quantity} how many kilogram/day ###.#
{dtype} dringtype: (0) beer (1) rice wine (2) wine (3) liquor (4) whisky #
{dyear} how many years have you drinked(y) ##
{tdquit} quit time: <mm/dd/yy>

a9 disease history:
{disease}Are you suffer from disease: (1)no (2)yes #

Disease 1:
{kind1} disease type ### disease {name1} __________
{dtime1} When did you get this disease? <mm/dd/yy>

{dunit1} Where did you get this diagnosis:
##
{dmeans1} diagnosing method: ##
{current1} current situation of this disease(1)cured (2)improvement(3)unrecovery (4)worse#
{remark1} remarks ##

Disease 2:
{kind2} disease type ### disease {name2}__________
{dtime2} When did you get this disease <mm/dd/yy>
{dunit2} Where did you get this diagnosis ##
{dmeans2} diagnosing method ##
{current2} current situation of this disease(1)cured (2)improvement(3)unrecovery (4)worse#
{remark2} remarks ##

Diseas 3:
{kind3} disease type ### disease {name3}__________
{dtime3} When did you get this disease <mm/dd/yy>
{dunit3} Where did you get this diagnosis: ##
{dmeans3} Diagnosing method: ##
{current3} current situation of this disease(1)cured (2)improvement(3)unrecovery (4)worse #
{remark3}remarks ##

{symptom1} present symptoms: ##
{symptom2} ## {symptom3} ## {symptom4} ##
{dose} Medicine (>monthes) ? (0) no (1) yes #
{remedy1} Name of medicine ##
{remedy2} ## {remedy3} ## {remedy4} ## {remedy5} ##
{defect} Inherited abnormality: (0) yes(1) no #
{defsex} in (1)male (2)female #
{defkind} What kind of abnormality ##

Reproductive Fuction(you or your wife):
menstruate history:
{menarche} menrache age(y): ##
{mperiod} menstruate cycle: (1)<25 days (2)25-35days (3)>=35days #
{mtime} menses duration: (1)<3 days (2)3-7days (3)>7 days #
{pain} dysmenorrhea (1)no (2)occasionaly (3)often #
{menopaus} menopausal age(year): ##
bearing history:
{tmarry} wedding time <mm/dd/yy>
{bear} Do you want to bear after your marriage: (0)no (1)yes #
{sterilit} Have you ever have no pregnant two yeas after marriage without contraception (0)no (1)yes #
{examine} if sterlity, did you go to see doctor for it (0)no (1)yes

#

{cause} accoding to doctor, the reason is in: (1)male (2)female (3)both (4)unawareness #

{femcause} if the problem in female, which problem: (1)ovary (2)oviduct (3)uterus (4)other problem (5)unawareness #

{mancause} if the problem in man, which problem: (1)testicle (2)varicosity (3)pudendum (4)sperm (5)other problem (6)unawareness #

{child} by far, how many children do you have? #

{boy} the number of boys # {girl}the number of girls #

{pregnan} how many times pregnancy did you have during your life; # {mabort} how many times of induced abortion #

{nabort} how many times spontaneous abortion; # {stillb} how many times of s0tillbirth; #

{total} total times of abortion #

{trest} how many month did you leave exposure after your pregnancy. ##

{btype}which worktype before your leaving ##

Partner:

{matename} name:______ {munit} unit: ## {mtype} job: ##

{moccu} occupation:(1)worker(2)farmer(3)staff(4)cardre(5)others #

{mage} age ##

{meduca} Education:
(0) illiterate (1) primary (2) middle (3) high school (4) master #

{mfactor} Exposed to: #

(1)pesticides (2)metal (3)organic sovents (4)Lab chemicaals (5)noise (6)radiation (7)medicine (8)others

{mdisease} Did she(he) suffer from chronic diseases: (0)no(1)yes #

{md1} What kind of disease ## {md2} ## {md3} ## {md4} ## {md5} ##

{msmoke} smoking (0)never (1)yes (2)stop smoking #

{tmsquit} When did you stop smoking? <mm/dd/yy>

{msyear} how many years have you smoked: ##

{mpackage} how many package of cigratte do you take every day: #

{mdrink} ing alcohol? (0) Never (1) occasionaly (2) often (3) quit #

{mdyear} How many year did you drinking alcohol ##

{mdquanti} how many kilogram/day ##

{tmdquit} when did you stop drinking <mm/dd/yy>

{mdose} Medicine (>monthes) ? (0) no (1) yes #

{mremedy1} which medicine do you often take ##

_

问卷表

一. 基本情况

编号　　　　　　　　　　骨密度测定编号

尿镉(ug/l)　　　　　　尿镉(ug/g.cr)

尿肌酐(g/l)

身高 (cm)　　　　　　体重　(Kg)　　　##.#

姓名______　　　　　　　　　　##

年龄　　　　　　　　　　　　　　　　　　　　##

性别:(1)男性 (2)女性　　　　　　　　　　　　　　#

教育程度：（0）无（1）小学（2）中学（3）高中（4）大专以上　　#

婚姻　(0)未婚　(1)离异　(2)已婚　(3)再婚　(4)丧偶　　#

出生地：是否当地出生(1)是　(0)否　　　　　　　　#

外迁：何时由外地迁入　　　　　　　　<mm/dd/yy>

职业史　　　　　　　　　　　　　　　#

危险因素:

何时开始该项工作　　　　　　　　　　<mm/dd/yy>

何时终止该项工作　　　　　　　　　　<mm/dd/yy>

工作单位　　　　　　##

工种　　　　　　　　##

暴露时间(h/day)　　　　　　　　　　　　　##

暴露水平(mg/m3)　　　　　　　　　　　　####

吸烟史：是否吸烟（0）否（1）是（2）戒烟　　　　#

何时戒烟　　　　　　　　　　　　　<mm/dd/yy>

何时开始吸烟　　　　　　　　　　　<mm/dd/yy>

吸烟量(包/天)　　　　　　　　##

烟草类型（0）商品烟（1）自制　　　　　　#

饮酒史　（0）从未（1）偶尔（2）经常（3）戒酒　　#

何时开始饮酒　　　　　　　　　　　<mm/dd/yy>

饮酒量（ml/d）　　　　　　　　　　　###.#

饮酒类型：（0）啤酒（1）米酒（2）白酒（3）黄酒（4）威士忌 #

饮酒时间（年）　　　　　　　　　　##

戒酒时间　　　　　　　　　　　　　<mm/dd/yy>

病史

是否患有疾病　　(1)否 (2)是　　　#

疾病 1:

疾病类型　###　　　疾病名字 __________

何时患病　<mm/dd/yy>

何处诊断:　　　　　　　　　　　　##

诊断方法:　　　　　　　　　　　　　　　##

疾病目前状态(1)治愈 (2)好转(3)未复发 (4)恶化#

疾病 2:

疾病类型　###　　　疾病名字 __________

何时患病　<mm/dd/yy>

何处诊断:　　　　　　　　　　　　##

诊断方法: ##

疾病目前状态(1)治愈 (2)好转(3)未复发 (4)恶化#

疾病 3:

疾病类型 ### 疾病名字 __________

何时患病 <mm/dd/yy>

何处诊断: ##

诊断方法: ##

疾病目前状态(1)治愈 (2)好转(3)未复发 (4)恶化#

目前症状: ##

是否服药（>monthes）？（0）否（1）是 #

药物名称 ##

遗传病史:（0）否(1）是 #

遗传疾病类型 ##

生殖功能(自己或配偶):

月经初潮年龄(y): ##

月经周期: (1)<25 天 (2)25-35 天 (3)>=天 #

月经持续时间: (1)<3 天 (2)3-7 天 (3)>7 天 #

痛经(1)无 (2 偶尔 (3)经常 #

绝经年龄: ##

生育史

结婚年龄 <mm/dd/yy>

是否打算生育: (0)否 (1)是 #

不孕不育史 (0)否 (1)是 #

原因: (1)丈夫 (2)妻子 (3)双方 (4)未知 #

如果是妻子的原因是以下哪种: (1)卵巢 (2)输卵管 (3)子宫 (4)其他 (5)未知 #

如果是男性原因是以下那种: (1)睾丸 (2)精索静脉 (3)生殖器(4)精子 (5)其他问题 (6)不明原因 #

你有几个孩子: #

男孩数量 # 女孩数量 #

怀孕次数 # 流产次数 #

自发流产次数 # 死产次数: #

总流产次数 #

怀孕后休假时间 ##

配偶情况:

姓名:______ 工作单位: ##

工作类型:(1)工人(2)农民(3)职员(4)公务员(5)其他 #

年龄 ##

受教育程度:(0) 无（1）小学（2）中学（3）高中（4）大专以上 #

职业接触史: #

(1)农药 (2)金属 (3)有机物 (4)实验室化学物 (5)噪声 (6)辐射 (7)药物 (8)其他

是否患有慢性疾病: (0)否 (1)是 #
疾病类型：## {md2} ## {md3} ## {md4} ## {md5} ##
吸烟史 (0)从未 (1)是 (2)戒烟 #
何时戒烟 <mm/dd/yy>
吸烟时间 ##
吸烟量（包/天）: #
饮酒史 （0）从未（1）偶尔（2）经常（3）戒酒 #
饮酒时间 ##
饮酒量（kg/d） ##
何时戒酒 <mm/dd/yy>
药物使用（>monthes）？（0）否（1）是 #
服用何种药物 ##
